# Detection of bifid mandibular condyle using computed tomography

**DOI:** 10.4317/medoral.17748

**Published:** 2012-05-01

**Authors:** Halil Sahman, Yildiray Sisman, Ahmet E. Sekerci, Elif Tarim-Ertas, Turgut Tokmak, Ibrahim S. Tuna

**Affiliations:** 1 DDS Department of Dentomaxillofacial Radiology, Faculty of Dentistry, Erciyes University, Kayseri, Turkey; 2 DDS, PhD Department of Dentomaxillofacial Radiology, Faculty of Dentistry, Erciyes University, Kayseri, Turkey; 3 DDS, PhD Department of Dentomaxillofacial Radiology, Faculty of Dentistry, Katip Celebi University, İzmir, Turkey; 4 MD Department of Radiology, School of Medicine, Erciyes University, Kayseri, Turkey; 5 MD Department of Radiology, Albert Einstein College of Medicine, New York, USA

## Abstract

Objective: To determine the frequency and characteristics of bifid mandibular condyle (BMC) using computed tomography (CT) evaluation. Study Design: A retrospective study was carried out using the CT records of 550 patients referred to the Medical School of Erciyes University (Kayseri, Turkey) between 2007 and 2010. T-tests were used to compare frequency of BMC between the left and right sides and between female and male patients. Statistical analysis was performed using SPSS software and a chi-squared test. Results: Of the 550 Patients, 10 patients (1.82%) were found to have BMCs. Five patients were female (50%) and five were male (50%). Of these 10 patients, 7 (70%) had unilateral and 3 (30%) had bilateral BMCs. As a result, a total of 13 BMCs were found in 10 patients. No statistically significant differences were found between either the right- and left-sided BMCs or between female and male patients (p >.05). Conclusions: To our knowledge, this is the first retrospective study investigating the prevalence and characteristics of BMC using computed tomography. Although BMC is an uncommon anomaly, it may be a more frequent condition in the Turkish population. Further studies and research on the orientation of duplicated condylar heads should be carried out.

** Key words:**Computed tomography, bifid condyle, double-headed condyle, orientation, frequency.

## Introduction

Bifid mandibular condyle (BMC), also known as “double-headed condyle”, is an uncommon anomaly which has an unknown etiology and uncertain pathogenesis (1,2). Most cases of BMCs are asymptomatic and generally diagnosed as an incidental finding during routine radiographic examinations (3,4). With the increased use of panoramic radiographs, investigations of the etiology and epidemiology of BMCs have increased. Although the exact etiology of BMC is unknown, some circumstances such as trauma, teratogenic drug use, genetic tendency, infection and exposure to radiation may be responsible for these variations (5,6). Other authors reported that BMC could be an embryological malformation (2,7). However, the most likely theory is traumatic origin such as the application of forceps during birth, condylar fracture resulting from an accident or surgical condylectomy (8-11).

The term “bifid” is derived from the Latin word meaning “cleft into two parts”. Hence, it is characterized by the duplication of the mandibular condyle and a groove between these two condylar heads. It has been reported that, bifid condyles can be oriented anteroposteriorly or mediolateraly and the rift between the duplicated condylar heads can be distinct or indistinct (1,12).

A review of the literature revealed 112 cases of bifid mandibular condyles in living subjects (11-15). Although it is reported that BMC is a rare condition, it is increasingly being detected due to the use of advanced imaging techniques, especially computed tomography (CT), cone beam CT (CBCT) and magnetic resonance imaging (MRI) (11,13,16). Tomographic techniques provide good opportunities for evaluating the temporomandibular joint (TMJ) region and disorders like BMC (10,17).

The goal of this study was to assess the frequency of BMC using CT. As a secondary goal, we aimed to examine the characteristics of bifid mandibular condyles from their CT images.

## Material and Methods

A retrospective study was performed using the CT records of 550 patients referred to the Medical School of Erciyes University from 2007 to 2010. The CT ima-ges were obtained due to patients’ previous medical problems. Exposure factors and slice thickness (0.625 mm – 1.25 mm) varied according to the requirements of each individual and images were obtained with a CT Machine (Light Speed GE Medical Systems, Milwaukee, Wisconsin, USA) by a radiology technician. Reformat images were generated in PACS (Picture Archiving and Communication Systems) and evaluated by two observers using Infinitt Software Version 3.0.8.1 (Infinitt Medical Imaging and Archive System, Seoul, South Korea). One of them was a medical radiologist with ten years of experience and the other was an oral radiologist who with six years of experience in CT and panoramic imaging. To ensure the accuracy of the diagnosis, only the cases that were confirmed by both observers as having BMCs were scored as present. Finally, 10 BMC cases found in the 550 patients were reexamined and approved by another oral radiologist (Y.S).

**Figure 1:**
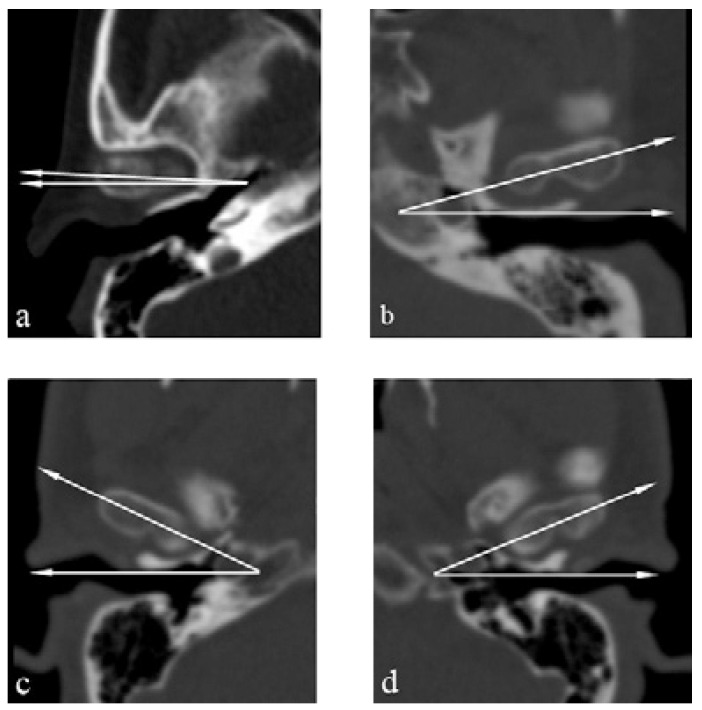
Axial CT scans of the patients revealed the following angles between the axis of the condyle and the transverse plane. a) 3º (Patient-5, right side), b) 15º (Patient-1, left side), c) 25º (Patient-6, right side), d) 22º (Patient-6, left side).

CT images were evaluated for the presence and features of bifid mandibular condyle. The cases of BMC observed in the 550 CT images were reviewed and analyzed in accordance with age, gender, side and angle of orientation ( Table 1). Orientation of the condyles was shown in axial CT section according to the angle between the axis of the condyle and the transverse plane (Fig. 1). Proper axial sections which include a clear appearance of the rift were selected. Axis of the condyle was drawn through the most extreme points of the condyle.

**Table 1:**
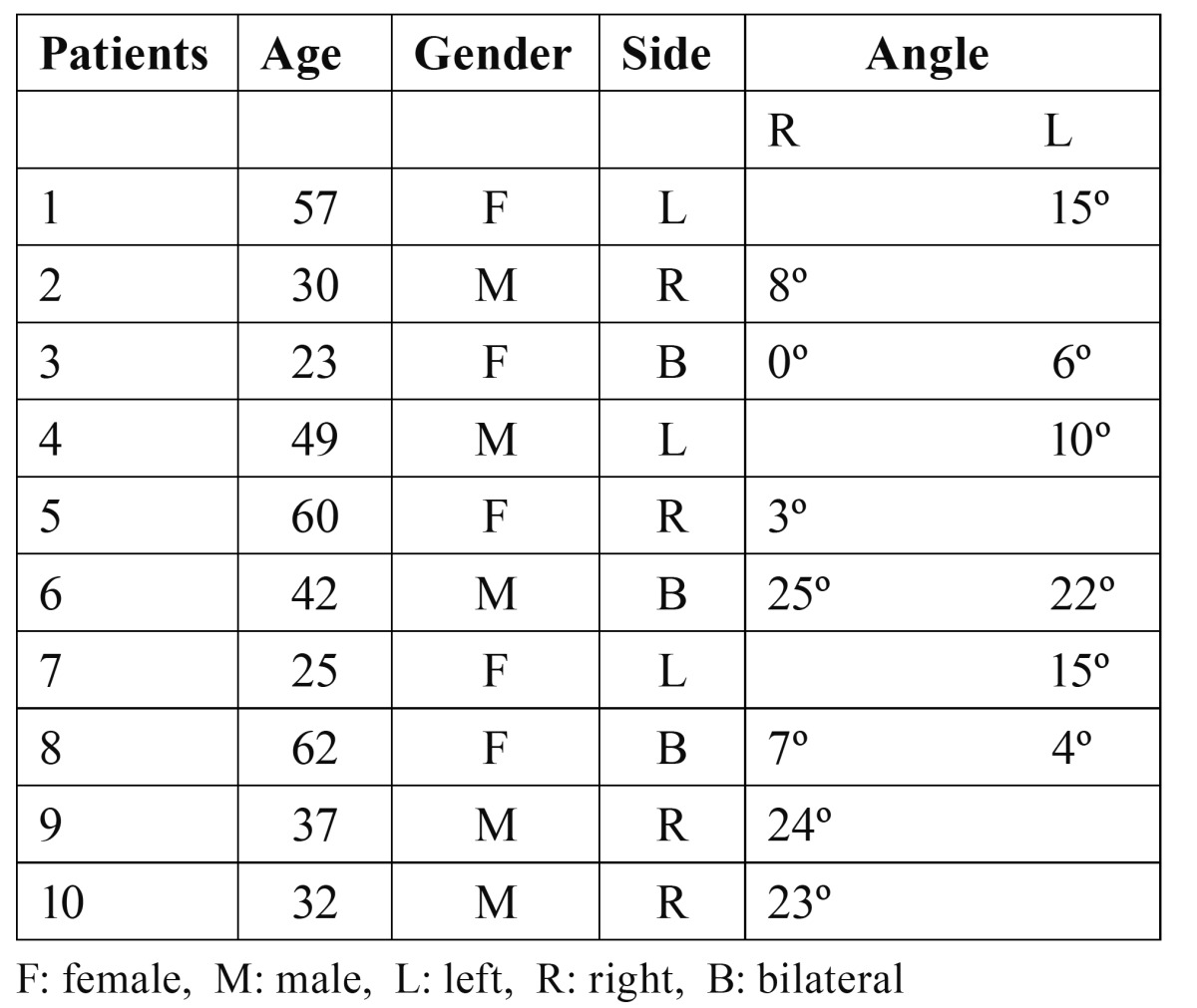
Bifid mandibular condyle in a Turkish patient population.

Of all the patients with findings of BMC, we were able to contact six of them by phone. We inquired about history of trauma and TMJ dysfunction such as clicking, crepitation, pain and limited mouth opening were asked.

The observed results were analyzed with SPSS 16.0 (Statistical Package for Social Science Inc., hicago, Illinois, USA). Chi-squared test was used to determine potential differences in the distribution of BMCs when stratified by gender and side. A p value of < 0.05 was considered statistically significant.

## Results

A total of 550 CT images obtained between 2007 and 2010 were evaluated. Of these patients, 328 (59.6%) were male and 222 (40.4%) were female (ages range 9 - 88 years; mean age 38.8 ± 15.5). Among these records, 13 bifid mandibular condyles were found in 10 (1.82%) patients. The frequency of BMC was found to be 1.52% in males and 2.25% in females with no significant gender difference found (p >.05). The ages of the patients ranged from 23 to 62 years (mean age 37.9 ± 12.3). Of these ten pa-tients, five were female (50%) and five were male (50%). Among them, seven (70%) of the BMCs were unilateral and three (30%) were bilateral. Four cases (57%) were on the right and three cases (43%) were on the left side with no significant side difference found (p >.05).

Of the six patients contacted, we learned that one had passed away four years ago and one was in prison. According to the anam-nesis of the remaining four patients, the following information was obtained: patient 7 had a history of head trauma due to a traffic accident and clicking on mouth opening; patient 8 also had a history of head trauma resulting from a traffic accident and bilateral TMJ pain; patient 9 and 10 complained only of clicking on mouth opening.

**Figure 2:**
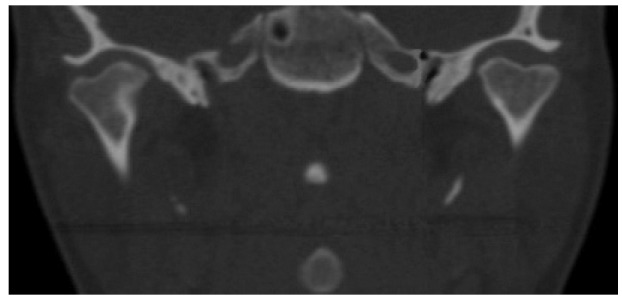
Coronal CT scan of the patient (Patient 3) revealed a bilateral bifid mandibular condyle.

**Figure 3:**
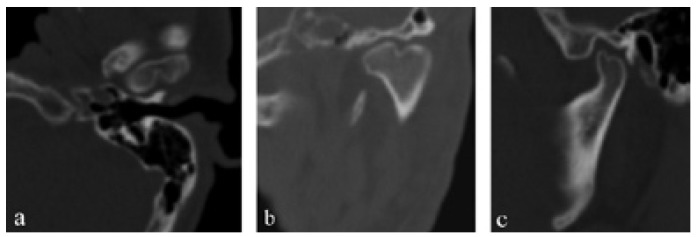
Axial a) coronal b) and sagittal c) CT scans (Patient-6) revealed left-sided BMC in a bilateral case.

**Figure 4:**
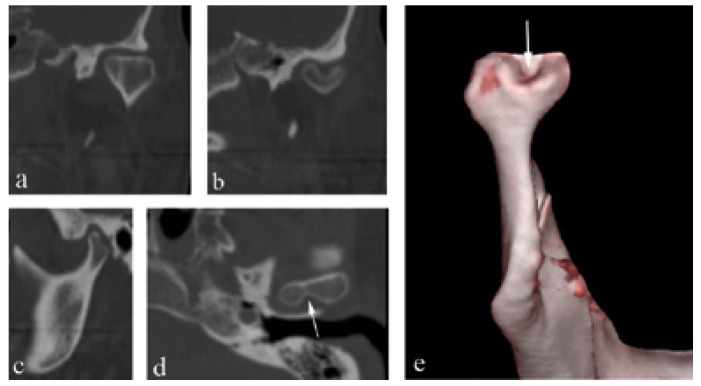
Coronal. a) CT scan of the patient (Patient-1) revealed no BMC. However, posterior slice, b) showed a clear BMC. Sagittal, c) CT scan showed irregular appearance of the posterior region of the condyle. Axial, d) CT scan showed a rift in the posterior of the condyle (arrow). 3D reconstruction image from posterior view, e) showed a partial BMC appearance and concavity between the duplicated condylar heads (arrow).

Figure 2 shows the coronal CT image of a bilateral BMC. Figure 3 shows CT images in three planes of a left-sided BMC. Figure 4 shows CT images in three planes and a 3D reconstruction of another case which had an appearance like partial BMC.

## Discussion

Bifid mandibular condyle is an uncommon entity usually discovered as an incidental finding during routine radiographic examinations (6). It is now being more frequently diagnosed and analyzed due to advanced imaging techniques, particularly CT, CBCT and MRI (11,13,16).

Hrdlicka first described bifid mandibular condyles in 1941. He found 21 examples in dried skulls in the Smithsonian Institution. Eighteen of these cases were unilateral and three were bilateral. In 1948, Schier (18) did the first study in a living subject and reported one case. Although numerous case reports have been reported, especially in the last decade, epidemiological studies in this field are limited. In a study carried out on nonliving subjects, Szenpetery et al. (19) reported seven (0.3%) cases of BMC in 1882 skulls with 2077 condyles. Only two epidemiological studies have been carried out on living subjects. In 2008, Menezes et al. (6) examined 50,080 panoramic radiographs in a Brazilian population and found only nine (0.018%) cases of BMC. Subse-quently, in 2010, Miloglu et al. (12) exami-ned 10,200 panoramic radiographs in a Turkish population and reported 32 (0.3%) cases of BMC. Both of these studies used panoramic radiographs and other conventional radiographic techniques. Hence, to our knowledge, this is the first retrospective study performed with computed tomography.

Our study presents 10 (1.82%) patients with BMC in 550 CT records obtained between 2007 and 2010. Compared to the pano-ramic based studies, considerable differences in the value of frequency were observed. Miloglu et al. (12) found a significantly higher frequency rate (0.3 %) than that in Menezes et al.’s study (0.018) (6). However, the frequency (1.82%) found in our CT-based study is six times higher than the frequency rate reported by Miloglu et al. (12). This significant difference between two studies in the same population may have occurred from misinterpretations of the panoramic radiographs.

In the literature, the occurrence of BMC does not show a predilection for any particular age group. In a study reported by Loh and Yeo (20), most of the patients were over 20 years old. In another study, eight of the nine patients with BMC were over 20 years old (6). These findings were consistent with the findings of Miloglu et al.’s (12) and our study (23 to 62, mean age 41.7). The authors believe that, BMC occurrence is not directly related with an increase in age. In our country, it might occur because the majority of patients referred to dental hospitals are adults and elders.

The occurrence of BMC also does not appear to show gender differences. In the literature, current reports revealed an average female-male ratio of 1.1:1 (a total of 112 cases of BMC, information was insufficient for 6 cases). Antoniades et al. (9) found a male-female ratio of 1.5:1, whereas Menezes et al. (6) found a significantly higher female-male ratio of 3.5:1. However, Mi-loglu et al. (12) reported a very similar ratio between genders (female-male, the ratio of 1.13:1) in a Turkish population and consistent with this finding, the female-male ratio of our study was also found to be identical (1:1). In this study, no statistically significant differences were found between female and male patients (p >.05).

A current literature review in living patients revealed a total of 112 cases of BMC (information was insufficient for 6 cases) (11-15). Of the 106 cases, 26 were bilateral and 80 were unilateral: 38 were on the right and 42 were on the left side (left–right, 1.1:1). The ratio of unilateral-bilateral cases in the literature was 3.1:1. In the present study, a ratio of 2.3:1 was observed, which is lower than that reported in the literature. However, the right-left ratio in this study was detected to be 1:1 which is very close to previous studies. In this study, no statistically significant differences were found between the right- and left-sided BMCs (p >.05).

The exact etiology of bifid mandibular condyle is unknown. However, the most suitable theory is trauma (21,22). Thomason and Yusuf (23) reported two cases of traumatic condyle fracture with subsequent unilateral BMC. Also, Antoniades et al. (21) presented a case of unilateral BMC which resulted following a sagittal condylar fracture. On the other hand, minor trauma to the growth center or deficient remodeling of the mandibular condyle may subsequently result in a variation such as BMC (21,24). In addition, TMJ ankylosis may cause the formation of BMC. Thus, in a retrospective study, Rehman et al. (11) reported 10 cases of BMC in 37 patients with TMJ ankylosis. Of those ten cases, nine were post-traumatic and one was post-infectious. Furthermore, Gulati et al. (15) reported two cases of BMC with joint ankylosis. Although, trauma is considered as the most common possible etiology, comparative studies have shown that the majority of patients had no history of previous trauma or TMJ complaints (9,12,20). In the present study, of the four patients contacted, two of them had a history of trauma, three of them had clicking on mouth opening and one of them had a history of pain. No other symptoms were described by the patients.

In the literature, two patterns of BMC have been reported. Condylar heads can be oriented anteroposteriorly (anteroposterior pattern) or mediolateraly (medio-lateral pattern) (12,25). However, we believe that this classification is not sufficient for all cases. For example, a BMC can be oriented in an oblique position, neither anteroposterior nor mediolateral. Thus, it is not possible to make a certain diagnosis about its exact pattern with conventional radiographic techniques. In this CT-based study, all of the cases were in the mediolateral orientation. However, the angles between the axis of the condyle and the transverse plane differed from 0º to 25º ( Table 1). We consider that, as the angle increases (a) duplicated condylar heads can be seen more clearly in sagittal CT sections (Fig. 3) and (b) duplicated condylar heads can have a superposed or anteroposterior position in panoramic radiographs. Thus, clinicians could misdiagnose the orientation of condyles in panoramic radiographs. In addition, the rift between the duplicated condylar heads may be partial, not overall, as in patient-1 (Fig. 4). These kinds of cases could be termed as “partial bifid condyle” and it is more difficult to diagnose with panoramic radiographs.

In general, TMJ dysfunction or pain is not evident in BMC cases. Hence, panoramic radiographs and other conventional radiographic techniques are sufficient in most cases. However, in patients with clinical symptoms, advanced imaging techniques such as CT, CBCT and MRI should be performed in order to support diagnosis and treatment planning. Tomographic techniques are the best choices for TMJ examination with the advance of three-dimensional visualization without superpositioning. In addition, MRI is considered as the gold standard for TMJ imaging as it allows visualization of soft tissue and surrounding particular structures to determine the exact pathology of TMJ (26). In recent years, CBCT applications have become widespread. Thus, in order to avoid excessive radiation, clinicians should prefer CBCT to other tomographic techniques.

In the light of these findings, we present the following conclusions with regard to bifid mandibular condyle (a) by the agency of imaging options in different planes and 3-D reformat, tomographic techniques like CT could represent bifid mandibular condyle and its orientation exactly. (b) Available classifications of BMC are inade-quate and further studies should be carried out in this field. (c) It is possible that BMC is a more frequent condition in the Turkish population.
